# Insulin Knowledge, Handling, and Storage among Diabetic Pilgrims during the Hajj Mass Gathering

**DOI:** 10.1155/2021/5596914

**Published:** 2021-05-28

**Authors:** Saber Yezli, Yara Yassin, Abdulaziz Mushi, Bander Balkhi, Anas Khan

**Affiliations:** ^1^The Global Centre for Mass Gatherings Medicine, Ministry of Health, Riyadh, Saudi Arabia; ^2^Department of Clinical Pharmacy, College of Pharmacy, King Saud University, Riyadh, Saudi Arabia; ^3^Department of Emergency Medicine, College of Medicine, King Saud University, Riyadh, Saudi Arabia

## Abstract

**Background:**

Diabetes is one of the most common underlying health conditions among Hajj pilgrims. Many diabetics manage their condition using insulin, which requires appropriate storage conditions to maintain its stability and effectiveness. We aimed to investigate insulin knowledge, storage, and handling among diabetic pilgrims during Hajj to identify specific areas for improvement.

**Method:**

Adult diabetic pilgrims from 22 countries were interviewed using a structured questionnaire during the 2019 Hajj.

**Results:**

The study enrolled 277 diabetic pilgrims with a mean age of 58.4 years (SD = 10.4, range: 20-83) and male : female ratio of 1.6 : 1. Most participants (86.4%) were literate and reported using insulin for a mean of 7.1 years (SD = 5.3, range: 1-23). Over 95% of pilgrims brought their insulin with them from their country of origin, where they also received most of their insulin storage information, mainly from physicians (77.8%) and pharmacists (59.6%). Pilgrims' knowledge regarding insulin storage was just above average (mean knowledge score = 0.51; SD = 0.23). Pilgrims who were literate and previously received education on insulin storage, those with a higher level of education, and those with a longer duration of insulin therapy, had significantly higher knowledge scores. Pilgrims' storage and handling of their insulin during Hajj also varied depending on the stages of their pilgrimage journey.

**Conclusion:**

Inadequate knowledge and inappropriate practices regarding insulin handling and storage were identified among diabetic Hajj pilgrims, which could compromise the quality of insulin and lead to health hazards. Improving diabetic pilgrims' knowledge of diabetes management, including insulin storage, will be beneficial during the pilgrimage and beyond.

## 1. Introduction

With an estimated global prevalence of 9.3% in 2019, diabetes is a significant global public health issue, responsible for sizeable mortality and morbidity worldwide and causing a substantial economic loss [1, 2]. The disease resulted in an estimated 1.37 million deaths in 2017 and cost an estimated US$1.31 trillion in 2015 [1, 3]. Diabetes can lead to kidney failure, blindness, and lower limb amputation and has also emerged as a leading cause of disability globally [4]. Poor glycemic control is a major risk factor for the development of diabetes complications and mortality and is directly linked to higher total healthcare, hospitalization, and medication costs [5, 6]. The management of diabetes is based on both medications and lifestyle modifications to achieve and maintain optimal glycemic control. Despite evidence that good diabetes management and glycemic control reduce microvascular and macrovascular complications [7], poor glycemic control is common among diabetics [8–11].

Although many diabetic patients use insulin therapy to manage their condition, lack of knowledge of the therapy and appropriate use of insulin is common and can lead to poor outcomes. Factors such as lack of adherence to therapy, incorrect insulin administration techniques, poor quality of insulin, inadequate insulin dose, and improper storage of the medication have been linked to poor glycemic control among diabetics [9–13]. Insulin is a labile drug, sensitive to extreme temperatures, sunlight, and shaking and hence needs to be appropriately handled and stored to be effective. Thus, insulin needs to be stored in refrigerators at 2-8°C (not frozen), almost always with a maximum usage/storage period of four weeks at standard room temperature (20-25°C) and away from sunlight [14, 15]. Improper storage of insulin decreases its potency and hence its pharmacological action and effectiveness, which may lead to failure to achieve optimal glycemic control amongst users [16]. Poor knowledge of insulin storage has been linked to inappropriate storage practices and poor glycemic control among diabetics [11, 12]. Ensuring optimal storage of insulin is particularly challenging for travelers as storage conditions and facilities may not be appropriate or available both during the travel journey and at the arrival destination.

Each year, over 2 million Muslim pilgrims travel to Saudi Arabia to perform the Hajj. Pilgrims originate from over 180 different countries with different socioeconomic, cultural, and health system backgrounds as well as varying levels of education, beliefs, and health literacy [17]. Many pilgrims are elderly and have underlying health conditions, particularly diabetes and hypertension [17, 18]. It is estimated that each year, up to 125,000 diabetics perform Hajj [18]. The Hajj journey presents specific challenges for pilgrims with diabetes, including in relation to insulin handling and storage [19, 20]. Many international pilgrims face numerous hours of flight, long transit times, and extended waiting times while traveling to KSA. During the Hajj, pilgrims are faced with physically demanding outdoor religious rites, changes in diets, and suboptimal hydration as well as hot temperatures (up to 50°C), which may impact their physiology, absorption of insulin, and interfere with its storage [20]. In particular, accessibility to appropriate storage facilities for insulin can be an issue for pilgrims during their Hajj journey [19]. These factors can impact insulin therapy among diabetic pilgrims and may negatively affect their wellbeing.

There is a lack of information regarding Hajj pilgrims' knowledge and practices relating to insulin storage and handling at the event. We aimed to investigate insulin knowledge, storage, and handling among diabetic pilgrims during the 2019 Hajj to identify specific areas for improvement.

## 2. Method

### 2.1. Study Design, Setting, and Population

A cross-sectional descriptive study, using a convenience sampling technique, was conducted among pilgrims in Makkah, Saudi Arabia, during the 2019 Hajj season. The study was performed from the 14^th^ to 19^th^ August 2019. Consenting adult pilgrims who declared that they were diabetic and using insulin were included in the study.

### 2.2. Data Collection Tools and the Scoring System

Data was collected via a specifically designed anonymous questionnaire based on available literature and tailored for the Hajj context. The questionnaire was reviewed by two experts and then piloted among 10 pilgrims before being finalized. The questionnaire collected demographic and health data as well as information regarding pilgrims' knowledge and practices relating to the handling and storage of insulin during their Hajj journey. The questionnaire was divided into three sections with questions regarding (1) pilgrims' characteristics (including age, gender, nationality, literacy and education levels, underlying health conditions, and duration of insulin therapy), (2) general knowledge regarding insulin handling and storage, and (3) insulin storage and handling during the Hajj pilgrimage.

A scoring system was used to score the knowledge responses as described previously [21]. Overall mean knowledge scores, ranging from 0 to 1, were calculated and further divided into 4 categories to reflect the level of knowledge among pilgrims. These were poor (score ≤ 0.25), below average (score > 0.25 to ≤0.50), above average (score > 0.5 − ≤0.75), and good (score > 0.75).

### 2.3. Statistical Analysis

Descriptive statistics such as mean and standard deviation (SD) were computed for quantitative variables, and frequencies and percentages were calculated for categorical variables. The association between categorical variables was evaluated by the chi-square, Mann–Whitney *U*, or Kruskal-Wallis tests as appropriate. Odds ratios with 95% confidence intervals (CIs) were computed to assess the presence and degree of association between the dependent versus independent variables. All tests for significance were two-sided, and *p* values < 0.05 were considered statistically significant. All analyses were performed using the SPSS 22.0 (SPSS Inc., Chicago, USA) software program.

### 2.4. Ethics and Confidentiality

Verbal consent was obtained from all participants. The study was approved by the King Fahad Medical City Ethics Committee and the Institutional Review Board. The questionnaire was anonymous and did not include any identifiers or personal information of the participants.

## 3. Results

### 3.1. Characteristics of the Study Population

The study enrolled 277 diabetic pilgrims from 22 countries, mainly from the Middle East and North Africa (MENA) region and South Asia (Table 1). Countries most represented were Pakistan (16.6%), Indonesia (12.6%), and Iraq (10.8%). Pilgrims were mostly male (61.7%) and had a mean age of 58.4 years (SD = 10.4, range: 20-83), and 84% were ≥50 years old. Most pilgrims (86.4%) were literate, with only 14.7% declaring not having a formal education. Hypertension was the most common comorbidity with diabetes (43.3%), followed by cardiovascular disease (6.8%). Pilgrims reported using insulin for a mean of 7.1 years (SD = 5.3, range: 1-23), with most (65.3%) using the medication for 10 years or less.

### 3.2. Knowledge of Insulin Storage among Pilgrims

Most pilgrims (83.6%) reported receiving some form of health education regarding the proper way to store insulin, nearly all of which was at their country of origin. Physicians and pharmacists were the main sources of information regarding insulin storage conditions (Table 2). Most pilgrims (>96%) were aware that temperature can affect insulin and that unused insulin had to be kept refrigerated (Table 2). However, a large proportion (60.6%) did not know how to visually tell if their insulin had gone bad, and less than half of the respondents knew the appropriate period insulin could be left at room temperature (Table 2).

**Figure 1:**
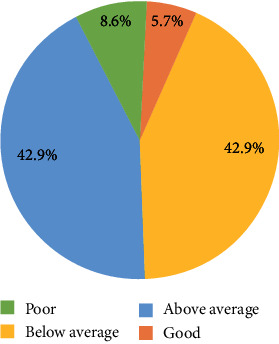
Level of insulin storage knowledge among diabetic Hajj pilgrims.

Insulin storage knowledge among diabetic pilgrims was just above average (overall mean knowledge score = 0.51; SD = 0.23), with only 8.6% considered to have good knowledge according to our criteria (Figure 1). There was a statistically significant difference in knowledge scores in relation to age, nationality, literacy, level of education, duration of insulin therapy, and receiving education on insulin storage (Table 3). Pilgrims who were literate and received education on insulin storage, those with a higher level of education, and those with longer duration of insulin therapy had significantly higher knowledge scores. Older pilgrims, on the other hand, had lower knowledge scores. No variable was significantly associated with good knowledge. However, the higher the level of education and the longer pilgrims have been taking insulin, the more likely they were to have good knowledge.

### 3.3. Insulin Storage and Handling among Pilgrims during the Hajj

Over 95% of diabetic pilgrims declared bringing their medication with them from their country of origin. Nearly 32% of respondents reported transporting their insulin to KSA inside their luggage without a cooling case, and most (86.3) discarded their used insulin syringes (needles) in regular trash (Table 2).

In contrast with the holy sites of Mina and Arafat, most pilgrims (>98%) reported that refrigerators were available at their accommodation while in Makkah and/or Madinah. Pilgrims' storage and handling of their insulin during Hajj varied depending on the stage of their pilgrimage journey (Table 4). The proportion of pilgrims who stored their insulin with their medical missions was significantly higher in the holy sites of Mina and Arafat compared to when pilgrims were staying in Makkah or Madinah (44.7% vs. 25.5%; *p* < 0.0001). Similarly, the use of cooling cases to store insulin was much higher in the holy sites of Mina and Arafat (60.9% vs. 29.1, *p* < 0.0001). While performing the Hajj rites, around 7% of pilgrims reported leaving their insulin at their accommodation. The use of water bottles to keep insulin at cooler temperatures was mainly used at the holy sites of Mina and Arafat (Table 4). Shared storage of insulin was uncommon among pilgrims (<4%).

## 4. Discussion

Certain aspects of the Hajj pilgrimage (e.g., travel, hot weather, outdoor religious activities, and variable accessibility to cool storage conditions) make optimal storage of insulin challenging for pilgrims with diabetes [19, 20]. We evaluated diabetic pilgrims' knowledge regarding insulin storage and their handling and storage of the medication during the 2019 Hajj. We found that most pilgrims brought their insulin with them from their country of origin, where they also received most of their insulin storage information, mainly from physicians and pharmacists. Pilgrims' knowledge regarding insulin storage was just above average and varied according to age, nationality, literacy, level of education, duration of insulin therapy, and receiving education on insulin storage. Pilgrims' storage and handling of their insulin during Hajj also varied depending on the stage of their pilgrimage journey.

Underlying health conditions are common among pilgrims, particularly diabetes and hypertension [18]. We report a high prevalence of comorbidity with hypertension among diabetic pilgrims, which may be explained by the common metabolic pathway among these two conditions [22]. Pilgrims using insulin have to take the medication regularly, including during travel. Therefore, it is unsurprising that most diabetic pilgrims in our study reported bringing their medication with them from outside KSA. Despite free healthcare provision for pilgrims during Hajj and the availability of medication during the event, it is prudent for pilgrims to bring their insulin with them in enough quantities to ensure they do not suddenly run out of the medication during Hajj. In general, most pilgrims with underlying health conditions bring their medication with them for Hajj, however, not always in sufficient quantities [23, 24].

Insulin is a labile, temperature-sensitive medication that is degraded by hydrolytic reactions or transformed to higher molecular weight components during storage and use. Hence, it is recommended that both unopened and opened insulin should be stored under specific temperature conditions for definite periods of time for optimum activity [14, 15]. Extreme temperatures should be avoided to prevent loss of potency, clumping, frosting, or precipitation. Visual inspection of insulin before injection is also important to ensure that the medication is not compromised or has not gone bad [14]. In the current study, most pilgrims were aware that insulin was temperature-sensitive and that unopen vials should be stored in a refrigerator. However, a large proportion did not know how to visually inspect their insulin or the recommended period insulin can be left at room temperature. These knowledge gaps can result in pilgrims injecting defective or less potent insulin and impacting their glycemic control.

Poor knowledge of diabetes and insulin therapy, including storage conditions, is a factor for poor glycemic control [9–13]. In our study, pilgrims had just above average knowledge regarding insulin storage, and only 8.6% were deemed to have a good level of knowledge. These results are in line with numerous other studies reporting that diabetic patients lack appropriate knowledge and practice regarding insulin therapy, including storage, handling, and disposal of the medication and its associated consumables [25–27]. Using a similar cut of criteria for good knowledge as ours (knowledge score > 0.75), studies from India and Ethiopia reported that only 4% and 31.3% of participants, respectively, had good knowledge regarding insulin therapy [25, 26]. Our results are concerning given that lack of insulin knowledge and inappropriate storage of the medication have been linked to poor glycemic control among diabetic patients [11, 12].

In the current study, pilgrims who were literate and previously received education on insulin storage, those with a higher level of education, and those with longer duration of insulin therapy had significantly higher knowledge scores. The longer the insulin therapy period, the more likely patients will be exposed to more information on how to better handle and store their insulin. Similarly, being educated enabled patients to understand and practice the written, oral, and any other means and directions and instructions of storage and handling better than illiterates. Level of education and insulin therapy duration were reported to have a significant effect on knowledge of insulin storage and handling among diabetic patients in other settings [25, 28]. Also, patients' education has been reported to increase their knowledge of diabetes and its management and to have a positive effect on metabolic control and patients' outcome [29, 30].

The Hajj journey involves stays in Makkah city and the holy sites of Mina and Arafat and may also include visits to the city of Madinah. How and where pilgrims chose to store their insulin varied depending on the pilgrimage journey. This may be a reflection of the differences in availability and accessibility to cool storage facilities at these locations. During their stay in Makkah and Madinah, most pilgrims reside in typical hotels, hence, did have access to a fridge. This was not the case in the holy sites of Mina and Arafat, where only a small proportion of pilgrims reported having access to a fridge. Pilgrims stay in these sites for only a few days, where they reside in large purposefully built tents; therefore, they may lack sufficient cool storage facilities. The above explains why most pilgrims in our study stored their insulin in refrigerators during their stay in Makkah and Madinah but turned to other means to keep their insulin at an appropriate temperature in Mina and Arafat—mainly utilizing their medical missions, which may have access to better storage facilities/conditions, or using personal cooling cases.

In areas with reduced access to fridges, we noted the use of alternative approaches by some pilgrims to keep their insulin at cool temperatures, including storage of insulin in water bottles. Such techniques have been reported among diabetics in limited-resource settings or during travel. In such instances, insulin was reported to be stored in water-filled clay pots, goatskin, vegetable gourds, or other traditional storage devices of various designs using evaporating cooling [31, 32]. Some of these techniques were reported to keep storage temperatures close to standard room temperature (20-25°C) even in hot climates [31]. However, these techniques can be a source of health hazards, and their use is generally discouraged [14]. For instance, keeping insulin in clay pots is likely to cause contamination as it is challenging to keep it clean. Similarly, insulin in use should never be kept immersed underwater as it carries a high risk of contamination, leading to loss of potency and the likelihood of causing injection abscesses [14].

Our study identified a number of practices among pilgrims that could compromise the overall quality of insulin and lead to health hazards. For example, a large proportion of pilgrims brought insulin to KSA in their standard luggage. When traveling by air, it is recommended that insulin is carried in the cabin, baggage, or handbags. Luggage that is checked in is stored in the aircraft's hold and may freeze, and any insulin in this luggage may lose its potency [14]. Also, we found that most pilgrims discarded their insulin needles and syringes in regular trash. Used sharps are a biomedical hazard as incorrect disposal could lead to needle-stick injuries, posing a risk of contracting blood-borne diseases [33]. Therefore, it is recommended that needles and lancets used by diabetic patients should be disposed of in designated sharps containers and not placed directly into the trash [14, 33].

Our results also show that a proportion of pilgrims left their insulin in their accommodations while performing the Hajj rituals. The latter can be physically demanding and may last many hours, during which pilgrims may require sustenance and access to their insulin. As such, we recommend that pilgrims should keep some insulin stored appropriately, accessible to them at all times during their Hajj journey to avoid practices that may negatively influence their health and diabetes management, such as skipping meals, having food without injecting insulin, and sharing of medication with other pilgrims. The latter is common among Hajj pilgrims [34].

Physicians followed by pharmacists were the primary sources of information regarding insulin storage among pilgrims. This is not surprising given that these healthcare providers are the main sources of insulin prescribing and dispensing as well as information on medication in general both among Hajj pilgrims and in the community [34, 35]. This emphasizes the importance of physicians and pharmacists in improving the knowledge and practice of patients regarding insulin handling and storage. A small proportion (<16%) of pilgrims reported obtaining information regarding their insulin storage from other sources such as the internet and family members, which is lower than found in another report [34]. However, such sources are not always reliable means of obtaining accurate medical information, and pilgrims should be aware of the risk of following directions on how to use, store, and dispose of medication obtained solely from such sources.

Health education for pilgrims regarding insulin handling and storage in general and specifically for the Hajj journey is crucial to improve their knowledge and practice. Notably, patients with diabetes need to be educated about temperature variations and duration of storage of insulin to maintain its efficacy [14]. Health education for diabetic patients should start at the country of origin and continue during the Hajj pilgrimage, led by physicians and pharmacists and the pilgrims' medical missions. Health education may also need to be extended to include these healthcare providers as studies have shown that insulin therapy knowledge and practice can be inadequate among this population as well [36, 37]. In addition, awareness of the facilities available for pilgrims in Hajj is important for pilgrims to prepare for the Hajj journey and ensure their medication will be stored and handled appropriately and if any adjustments are needed, including changes in dosage, storage, or frequency of replacement. The latter is particularly relevant for Hajj pilgrims as insulin stored at “room” temperature in hot climates loses its potency earlier than four weeks. In one study, insulin showed loss of potency at 32°C and 37°C temperatures after only three weeks. The authors concluded that in countries where the environmental temperature is often higher than 25°C, when storage cannot be assured at cool temperatures, insulin vials may be used only for 2 weeks after opening [16].

Our study has some limitations. The sample size, the sampling methodology, and the potential for volunteer bias may limit the generalizability of the findings. Also, data were collected using a questionnaire; therefore, responses obtained were prone to information bias. Finally, information regarding practice was self-reported and was not directly observed; therefore, the results may not accurately reflect actual practice among pilgrims.

In conclusion, we investigated insulin knowledge, handling, and storage among diabetic pilgrims during the Hajj mass gathering. While most pilgrims were aware that insulin is a temperature-sensitive medication, their knowledge regarding insulin storage was just above average. A number of poor practices were also identified that could compromise the quality of insulin and present health hazards for the users and others. Health education and awareness for pilgrims and provision of needed storage facilities during the Hajj are important to improve patient knowledge and practice in relation to their insulin therapy and avoid adverse health outcomes. Lessons learned from this study can also be applied to ensure appropriate storage and handling of other temperature-sensitive medications used by diabetics and other pilgrims [38]. Hajj is a unique opportunity to improve pilgrims' knowledge and management of their underlying health conditions and positively impact global public health.

## Figures and Tables

**Table 1:** Characteristics of the study population.

Variable	*N*	%
Total participants	277	
Gender		
Male	169	61.7
Female	105	38.3
Age (years)		
Mean, SD (range)	58.4, 10.4 (20-83)	
<35	8	2.9
35-49	36	13.1
50-64	154	56.0
≥60	77	28.0
Nationality		
Middle East and North Africa^∗^	110	40.1
South Asia	70	25.5
Sub-Saharan Africa	32	11.7
Southeast Asia	56	20.4
USA	6	2.2
Literacy		
Yes	209	86.4
No	33	13.6
Level of education		
University/higher education	94	37.3
Secondary education	74	29.4
Primary education	47	18.7
No formal education	37	14.7
Duration of insulin therapy		
≤5 years	72	47.1
6-10 years	39	26.5
>10 years	37	26.5
Underlying health conditions		
Hypertension	120	43.3
Cardiovascular disease	19	6.8
Chronic kidney disease	4	1.4
Chronic lung disease	6	2.1
Chronic liver disease	2	0.7
Immunosuppressive illness	1	0.3
Cancer	5	1.8

^∗^Including Turkey. SD: standard deviation; USA: United States of America.

**Table 2:** Insulin knowledge and practice among diabetic pilgrims.

Questions	Answers	*N*	%
*Knowledge*			
Can insulin be affected by storage temperature?			
	Yes	174	99.4
	No	1	0.6
Appropriate way to store unused insulin			
	Refrigerator	175	96.2
	At room temperature	16	8.8
	Do not know	1	0.5
How long can insulin be left at room temperature?			
	1 day	40	23.0
	Up to 1 week	0	0.0
	Up to 4 weeks	85	48.9
	Up to 6 months	0	0.0
	Do not know	49	28.2
How can you tell if insulin has gone bad?			
	Turns cloudy	59	33.7
	Shows traces of crystals	19	10.9
	Do not know	106	60.6
*Practice*			
How did you bring your insulin to KSA?			
	In my luggage (without cooling case)	58	31.9
	In the cooling case (in the luggage/carry on)	93	51.1
	Through medical mission	22	1.8
	Obtained it in Saudi Arabia	2	0.2
Source of insulin storage directions			
	Physician	133	77.8
	Pharmacist	102	59.6
	Label	9	5.3
	Internet	14	8.2
	Other	13	7.6
Where do you dispose of your insulin syringes (needles)?			
	Regular trash	151	86.3
	Specific disposal container	23	13.1
	Other	1	0.6

KSA: Kingdom of Saudi Arabia.

**Table 3:** Variables and insulin storage mean knowledge scores among diabetic pilgrims.

Variable	*N*	Mean	SD	*p* value
Gender				
Male	107	0.50	0.29	0.720
Female	67	0.50	0.22	
Age				
<35	6	0.60	0.15	<0.001
35-49	25	0.69	0.24	
50-64	104	0.47	0.19	
≥60	39	0.44	0.20	
Nationality				
Middle East and North Africa^∗^	63	0.49	0.20	0.008
South Asia	49	0.41	0.14	
Sub-Saharan Africa	15	0.53	0.18	
Southeast Asia	41	0.59	0.27	
United States of America	5	0.55	0.22	
Literacy				
Yes	133	0.53	0.21	0.002
No	20	0.37	0.16	
Level of education				
University/higher education	58	0.59	0.20	0.011
Secondary education	52	0.52	0.23	
Primary education	33	0.45	0.16	
No formal education	22	0.34	0.15	
Duration of insulin therapy				
≤5 years	72	0.44	0.18	0.009
6-10 years	39	0.52	0.18	
>10 years	37	0.55	0.24	
Education on insulin storage				
Yes	143	0.52	0.21	0.001
No	28	0.38	0.19	

^∗^Including Turkey. *p* value for the Mann–Whitney *U* or Kruskal-Wallis tests. SD: standard deviation.

**Table 4:** Insulin handling and storage during the Hajj.

	Makkah, *n* (%)	Madinah, *n* (%)	Mina, *n* (%)	Arafat, *n* (%)
Availability of a refrigerator	181 (100)	170 (97.7)	78 (43.1)	58 (32.8)
Storage of insulin during Hajj				
(i) With medical mission	49 (26.8)	44 (25.0)	84 (46.4)	79 (43.6)
(ii) In shared storage	7 (3.8)	5 (2.8)	5 (2.8)	5 (2.8)
(iii) With self	152 (83.1)	148 (84.1)	116 (64.1)	119 (65.7)
In refrigerator	108 (71.5)	107 (73.8)	17 (14.8)	18 (15.3)
In a cooling case	47 (31.1)	39 (26.9)	72 (62.6)	70 (59.3)
In a bottle of water	0	0	11 (9.5)	13 (11.0)
At room temperature	5 (3.3)	5 (3.4)	16 (13.9)	18 (15.3)
Storage of insulin while performing the Hajj rituals				
(i) With medical mission	50 (30.5)	44 (28.0)	87 (48.6)	80 (44.9)
(ii) In shared storage	3 (1.8)	3 (1.9)	6 (3.4)	6 (3.4)
(iii) Leave in accommodation	13 (7.9)	11 (7.0)	15 (8.4)	12 (6.7)
(iv) With self	134 (81.7)	129 (82.2)	114 (63.7)	118 (66.3)
In refrigerator	83 (62.4)	80 (62.5)	10 (8.7)	11 (9.2)
In a cooling case	49 (36.8)	46 (35.9)	78 (67.8)	78 (65.5)
In a bottle of water	1 (0.8)	0	9 (7.8)	10 (8.5)
At room temperature	7 (5.3)	5 (3.9)	17 (14.8)	18 (15.1)

## Data Availability

The datasets analyzed during the current study are not publicly available but are available from the corresponding author on reasonable request.

## References

[1] GBD Causes of Death Collaborators (2017). Global, regional, and national age-sex-specific mortality for 282 causes of death in 195 countries and territories, 1980-2017: A systematic analysis for the global burden of disease study. *The Lancet*.

[2] Saeedi P., Petersohn I., Salpea P. (2019). Global and regional diabetes prevalence estimates for 2019 and projections for 2030 and 2045: Results from the International Diabetes Federation Diabetes Atlas, 9^th^ edition. *Diabetes Research and Clinical Practice*.

[3] Bommer C., Heesemann E., Sagalova V. (2017). The global economic burden of diabetes in adults aged 20-79 years: a cost-of-illness study. *The Lancet Diabetes and Endocrinology*.

[4] GBD Disease Injury Incidence Prevalence Collaborators (2017). Global, regional, and national incidence, prevalence, and years lived with disability for 354 diseases and injuries for 195 countries and territories, 1990-2017: A systematic analysis for the global burden of disease study. *The Lancet*.

[5] Mata-Cases M., Rodríguez-Sánchez B., Mauricio D. (2020). The association between poor glycemic control and health care costs in people with diabetes: a population-based study. *Diabetes Care*.

[6] Kayar Y., Ilhan A., Kayar N. B. (2017). Relationship between the poor glycemic control and risk factors, life style and complications. *Biomedical Research*.

[7] UK Prospective Diabetes Study (UKPDS) Group (1998). Intensive blood-glucose control with sulphonylureas or insulin compared with conventional treatment and risk of complications in patients with type 2 diabetes (UKPDS 33). UK Prospective Diabetes Study (UKPDS) Group. *The Lancet*.

[8] Alzaheb R. A., Altemani A. H. (2018). The prevalence and determinants of poor glycemic control among adults with type 2 diabetes mellitus in Saudi Arabia. *Diabetes, Metabolic Syndrome and Obesity: Targets and Therapy*.

[9] Dedefo M. G., Abate S. K., Ejeta B. M., Korsa A. T. (2020). Predictors of poor glycemic control and level of glycemic control among diabetic patients in West Ethiopia. *Annals of Medicine and Surgery*.

[10] Angamo M. T., Melese B. H., Ayen W. Y. (2013). Determinants of glycemic control among insulin treated diabetic patients in Southwest Ethiopia: hospital based cross sectional study. *PLoS One*.

[11] Altebainawi A. F., Alrashidi M. N., Aljbreen M. K. (2020). Association of medication storage with diabetes control: a cross-sectional study from Saudi Arabia. *Saudi Pharmaceutical Journal*.

[12] Kituzi E. K., Karimi P. N., Nyamu D. G., Tirop I. J. (2016). Effect of insulin storage and administration methods on long term glycaemic control among adult diabetic patients in a Kenyan referral hospital. *East and Central African Journal of Pharmaceutical Sciences*.

[13] Romakin P., Mohammadnezhad M. (2019). Patient-related factors associated with poor glycaemic control among patients with type 2 diabetes mellitus. *Australian Journal of General Practice*.

[14] Bahendeka S., Kaushik R., Swai A. B. (2019). EADSG guidelines: insulin storage and optimisation of injection technique in diabetes management. *Diabetes Therapy*.

[15] Grajower M. M. (2014). How long can a vial of insulin be used after it is started: where are we 10 years later?. *Endocrine Practice*.

[16] Vimalavathini R., Gitanjali B. (2009). Effect of temperature on the potency & pharmacological action of insulin. *The Indian Journal of Medical Research*.

[17] Yezli S., Elganainy A., Awam A. (2018). Strengthening health security at the Hajj mass gatherings: a Harmonised Hajj Health Information System. *Journal of Travel Medicine*.

[18] Yezli S., Mushi A., Almuzaini Y., Balkhi B., Yassin Y., Khan A. (2021). Prevalence of diabetes and hypertension among Hajj pilgrims: a systematic review. *International Journal of Environmental Research and Public Health*.

[19] Ibrahim M., Abdelaziz S. I., Almagd M. A. (2018). Recommendations for management of diabetes and its complications during Hajj (Muslim pilgrimage). *BMJ Open Diabetes Research & Care*.

[20] Algeffari M. (2019). Diabetes and Hajj pilgrims: a narrative review of literature. *The Journal of the Pakistan Medical Association*.

[21] Alotaibi B., Yassin Y., Mushi A. (2019). Tuberculosis knowledge, attitude and practice among healthcare workers during the 2016 Hajj. *PLoS One*.

[22] Cheung B. M., Li C. (2012). Diabetes and hypertension: is there a common metabolic pathway?. *Current Atherosclerosis Reports*.

[23] Alqahtani A. S., Althimiri N. A., Bin Dhim N. F. (2019). Saudi Hajj pilgrims' preparation and uptake of health preventive measures during Hajj 2017. *Journal of Infection and Public Health*.

[24] BaDawood A. O., Bossei A. A., AlSabhani M. F., AlAhmari S. M., Shata M. T., Hamam A. F. (2020). The burden on EDs during Hajj due to pilgrim noncompliance with treatment for chronic conditions. *Saudi Journal of Emergency Medicine*.

[25] Netere A. K., Ashete E., Gebreyohannes E. A., Belachew S. A. (2020). Evaluations of knowledge, skills and practices of insulin storage and injection handling techniques of diabetic patients in Ethiopian primary hospitals. *BMC Public Health*.

[26] Priscilla S., Malarvizhi S., Das A. K., Natarajan V. (2019). The level of knowledge and attitude on insulin therapy in patients with diabetes mellitus in a teaching hospital of southern India. *Journal of Family Medicine and Primary Care*.

[27] Yilmaz U. D., Tarhan S. (2017). Determination of attitude and knowledge of type 2 diabetic patients towards insulin therapy in northern Cyprus. *The Journal of the Pakistan Medical Association*.

[28] Das Choudhury S., Das S. K., Hazra A. (2014). Survey of knowledge-attitude-practice concerning insulin use in adult diabetic patients in eastern India. *Indian Journal of Pharmacology*.

[29] Brown S. A. (1990). Studies of educational interventions and outcomes in diabetic adults: a meta-analysis revisited. *Patient Education and Counseling*.

[30] Vyas C., Dalal L., Talaviya P., Saboo B. (2017). Multiple educational programs improves glycemic control, quality of life with diminishing the impact of diabetes in poorly controlled type 1 diabetics. *Diabetes and Metabolic Syndrome: Clinical Research and Reviews*.

[31] Ogle G., Abdullah M., Mason D., Januszewski A., Besançon S. (2016). Insulin storage in hot climates without refrigeration: temperature reduction efficacy of clay pots and other techniques. *Diabetic Medicine*.

[32] Gill G., Price C., English P., Eriksson-Lee J. (2002). Traditional clay pots as storage containers for insulin in hot climates. *Tropical Doctor*.

[33] Satterfield D., Kling J. (1991). Diabetes educators encourage safe needle practice. *The Diabetes Educator*.

[34] Haseeb A., Faidah H. S., Elrajjal M. E. (2018). Pilgrims attitude and believes towards medications use during Hajj 2013. *18th Scientific Forum for the Research of Hajj, Umrah and Madinah Visit - Scientific Bulletin for 1439AH (2018)*.

[35] Hailat M., Al-Shdefat R. I., Muflih S. M. (2020). Public knowledge about dosage forms, routes of drug administration and medication proper storage conditions in Riyadh District, Saudi Arabia. *Journal of Pharmaceutical Health Services Research*.

[36] Robb A., Reid B., Laird E. A. (2017). Insulin knowledge and practice: a survey of district nurses in Northern Ireland. *British Journal of Community Nursing*.

[37] Derr R. L., Sivanandy M. S., Bronich-Hall L., Rodriguez A. (2007). Insulin-related knowledge among health care professionals in internal medicine. *Diabetes Spectrum: A Publication of the American Diabetes Association*.

[38] Hatchett R. (2017). The medicines refrigerator and the importance of the cold chain in the safe storage of medicines. *Nursing Standard*.

